# Review of direct impact health indicators of COVID-19 in the scientific literature published between January 2020 and June 2021: Cesar Garriga

**DOI:** 10.1093/eurpub/ckac129.061

**Published:** 2022-10-25

**Authors:** C Garriga, T Valero, A Diaz, C Rodriguez-Blazquez, MJ Forjaz, PHIRI WP5.1collaboration group

**Affiliations:** 1 National Centre for Epidemiology, Institute of Health Carlos III, Madrid, Spain; 2 Department of Epidemiology and Public Health, Sciensano, Brussels, Belgium

## Abstract

**Background:**

The Joint Action Population Health Information Research Infrastructure (PHIRI) seeks to create infrastructures to generate quality data on the COVID-19 pandemic between European countries. The aim of this study is to present a synthesis of health indicators used to evaluate the direct impact of COVID-19

**Methods:**

Scoping review using a common search strategy in Pubmed, Embase and WHO Covid-19 databases. Health indicators of direct impact of COVID-19 were obtained from observational studies in the general population, hospitals and long-term care facilities from papers published worldwide in English between 01/01/2020 and 06/31/2021. Titles and abstracts were screened first by 15 reviewers using the Rayyan tool. Any discrepancies were solved by agreement between reviewers. Then, articles containing indicators of direct impact were selected in a full-text reading phase. Of them, a random sample of 35 was drawn and their indicators were described.

**Results:**

After eliminating 262 duplicates 3891 records were reviewed. Screening discarded 3171 abstracts. Of 720 articles sought for retrieval, 445 met inclusion criteria for indicators extraction. In a sample of 35 papers (8.1%), 116 direct impact indicators of COVID-19 were identified. 28 morbidity indicators were found, classified as indicators of prevalence (n = 15), incidence (6), transmissibility (4) and underreported infection (4); 32 of mortality (mortality rate, 9; case fatality rate, 17; time to death, 2); and 54 for severity (complications, 27; mechanical ventilation, 12; hospitalization, 8; requiring ICU admission, 1; time from hospitalization to ICU admission, 1). Two composite indicators of severity and mortality were also identified.

**Conclusions:**

According to the scientific literature, a wide variety of health indicators has been used to measure the direct impact of COVID-19. The systematization of indicators used in the current COVID-19 pandemic could help for future health crises management.

